# Host Immune Responses to SARS-CoV-2 Vaccination in Northern Mexico: Structural Biology Insights and the Impact of Obesity

**DOI:** 10.3390/ijms27104319

**Published:** 2026-05-12

**Authors:** Carlo F. Medina-Ramírez, Jose L. Chavelas-Reyes, Josefina G. Rodríguez-González, Nadia A. Fernández-Santos, Lihua Wei, Francisco J. Cabrera-Santos, Eli J. Fuentes-Chávez, Luis M. Rodríguez-Martínez, Mario A. Rodríguez Pérez

**Affiliations:** 1 Instituto Politécnico Nacional, Centro de Biotecnología Genómica, Reynosa 88710, Mexico; cmedinar2100@alumno.ipn.mx (C.F.M.-R.);; 2 Universidad Autónoma de Coahuila, Centro de Estudios e Investigaciones Interdisciplinarios, Saltillo 25280, Mexico

**Keywords:** SARS-CoV-2, COVID-19, Mexico, obesity, metabolic disorder, immune response, structural biology

## Abstract

Understanding the molecular mechanisms underlying host immune responses to SARS-CoV-2 vaccination remains essential, particularly in populations with a high prevalence of obesity. In this cross-sectional study, we evaluated whether body mass index (BMI) is associated with vaccine-induced humoral immunity in a cohort from northeastern Mexico and discuss the findings within a structural immunology framework of spike antigenicity and antibody–epitope interactions. A total of 138 adults were recruited in Reynosa and Matamoros (June 2021–June 2022) and categorized as healthy weight, overweight, or obese according to BMI criteria. Serum anti-SARS-CoV-2 IgG was assessed using an ELISA-based assay, and differences across BMI groups were tested using the Kruskal–Wallis approach. Among all participants, 33.3% were classified as obese and 99.3% (137/138) were seropositive for anti-SARS-CoV-2 IgG. No significant differences in IgG levels were detected between BMI categories (*p* = 0.20). These results indicate that, in this Mexican cohort—sampled during a period of heterogeneous and often incomplete vaccination schedules—obesity was not associated with reduced detectable anti-SARS-CoV-2 IgG responses. Our findings support the need to integrate population-level serology with mechanistic studies that interrogate antibody quality (e.g., neutralization potency and epitope specificity) to better connect clinical determinants such as obesity with molecular correlates of protection.

## 1. Introduction

Obesity (BMI ≥ 30 kg/m^2^) is a metabolic condition marked by excessive accumulation of adipose tissue, which induces a chronic inflammatory state capable of impairing both innate and adaptive immune responses. As a result, individuals with obesity are more vulnerable to infections and their related complications compared to individuals with normal weight [1].

Obesity represents the leading public health challenge in Mexico and has shown a steady increase over the past three decades. Currently, 36.1% of Mexican adults meet the criteria for obesity (BMI ≥ 30 kg/m^2^), with prevalence notably higher among women than men (40.2% vs. 30.5%) [2,3].

Adipose tissue may function as an enhancer of viral dissemination, intensifying immune activation and amplifying the cytokine cascade [4]. Vaccination is considered the most effective tool to control the spread of new SARS-CoV-2 variants [5]. Studies have shown that IgG antibodies against SARS-CoV-2 antibody responses are negatively correlated with body mass index (BMI) in obese individuals, according to current evidence on the impact of obesity on humoral immunity [6].

Although individuals with obesity may exhibit an initial protective response following vaccination, several studies have shown that this protection tends to decline more rapidly over time, suggesting reduced vaccine effectiveness and impaired maintenance of immunological memory [7]. Neutralizing IgG antibodies against the Spike protein provide essential protection by blocking virus-ACE2 receptor interactions and preventing infection. However, individuals with obesity produce fewer neutralizing antibodies and a higher proportion of non-neutralizing antibodies that recognize viral antigens but fail to inhibit infection [8]. Strong evidence of an association between severe obesity and an accelerated decline in humoral immunity with SARS-CoV-2 vaccines has been observed in different studies despite an initial adequate early humoral and cellular immune response [1]. Individuals with obesity produce neutralizing antibodies of lower quantity and quality. The chronic pro-inflammatory state alters immune function, impairing antibody generation and their subsequent structural interaction with viral surfaces [8].

Recognition of antibodies using a structural approach is crucial as it provides detailed information on how modifications in the structure of antibodies can influence their efficacy [9]. Understanding these variations is essential to improve vaccination strategies, especially in populations with a high prevalence of obesity. The structural alterations observed in antibodies from obese individuals may affect vaccine efficacy, suggesting the need to design vaccines that consider these differences to achieve optimal protection [10].

Therefore, to better understand the molecular mechanisms underlying host immune responses and the rationale of mass vaccination campaigns, this study evaluates the generation of anti-SARS-CoV-2 IgG antibodies in a cohort from northern Mexico categorized by BMI. Furthermore, we interpret our serological findings through a structural biology framework, discussing how the conformational dynamics of the Spike protein and epitope accessibility might interact with the immunometabolic alterations caused by obesity.

## 2. Results

**Figure 1:**
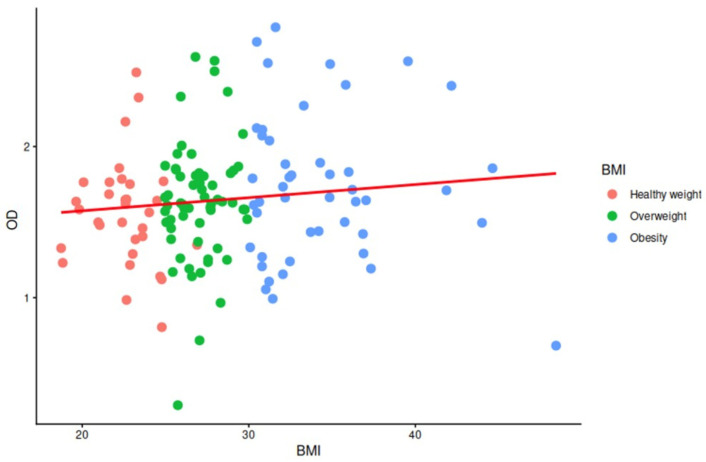
Association between body mass index and optical density values. Scatter plot showing the relationship between BMI and optical density (OD) values. Individuals were classified into three BMI categories: Healthy weight, Overweight, and Obesity. Data points are colored according to BMI group, and the red line represents the fitted linear regression trend across all observations.

A total of 138 serum samples were collected and analyzed from individuals residing in northeastern Mexico who had received at least one dose of a SARS-CoV-2 vaccine. At the time of sampling, the complete vaccination schedule was not yet widely available in the region, and therefore participants were considered partially vaccinated. Of the total samples evaluated, 137/138 (99.3%) yielded positive ELISA results, indicating detectable anti-SARS-CoV-2 IgG in nearly all individuals examined. The mean BMI was 25.8 kg/m^2^, with a median of 27.1 kg/m^2^ (range: 17.2–48.4 kg/m^2^). Most participants were classified as overweight (43.5%), followed by obesity (33.3%) and healthy weight (23.2%) (Figure 1). Kruskal–Wallis analysis showed no significant differences in OD values among BMI categories (*p* = 0.20). Because the normality assumption was not met after transformation (Shapiro–Wilk W = 0.96, *p* < 0.0028), non-parametric analysis was retained (Table 1).

**Figure 2:**
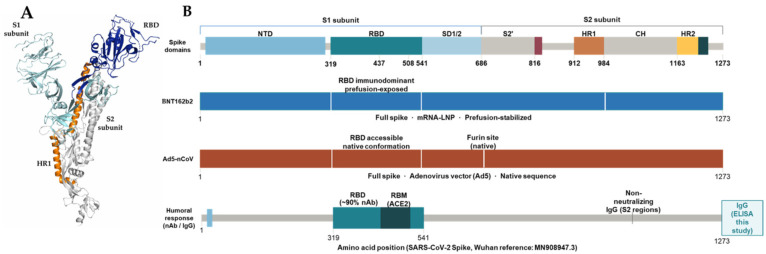
Structural organization of the SARS-CoV-2 spike protein and antigenic coverage of the predominant vaccines administered in this cohort. (**A**) Three-dimensional ribbon representation of the prefusion SARS-CoV-2 spike protein trimer. (**B**) Schematic linear domain organization of the spike protein showing the structural and functional regions targeted by BNT162b2 and Ad5-nCoV vaccine platforms and the corresponding humoral immune response.

In this study, 138 individuals were analyzed. Among individual received the Ad5-nCoVvaccine, of which 56 (100%) individuals tested positive, 54 individuals received the BNT162b2 vaccine of which 53 (99%) tested positive, 28 individuals received other vaccines (CoronaVac, ChAdOx1-S, BNT162b2, Gam-COVID-Vac, and mRNA-1273) where all the samples tested positive (Table 2). Vaccinated individuals with Ad5-nCoVshowed a widespread range of OD values (1.05–2.57), the BNT162b2 vaccine showed a range of OD values (0.29–2.69) and finally, individuals with other vaccines (CoronaVac, ChAdOx1-S, Gam-COVID-Vac, and mRNA-1273) showed a range of OD values (range 0.72–2.79) (Figure 2). Other Mexican cohorts have also documented variable optical density (OD) readings among vaccinated individuals. For example, participants immunized with the Ad5-nCoVvaccine in Oaxaca exhibited OD values ranging from 0.26 to 2.24, while those who received ChAdOx1-S presented values between 0.71 and 1.79. In comparison, individuals vaccinated with other platforms, including CoronaVac, BNT162b2, Gam-COVID-Vac, and mRNA-1273, showed OD readings within the range of 1.12 to 1.99, reflecting heterogeneity in antibody responses across different vaccine types [11]. In addition, data from another Mexican cohort reported the median levels of neutralizing antibody inhibition following the completion of full vaccination schedules with different platforms.

The recorded values were 97.23% for BNT162b2, 97.61% for mRNA-1273, 97.23% for Ad5-nCoV, and 97.18% for ChAdOx1-S, indicating a strong neutralizing capacity across these vaccines. By contrast, individuals immunized with CoronaVac exhibited a substantially lower median inhibition value of 74.05%, highlighting marked differences in the magnitude of neutralizing antibody responses depending on the vaccine administered [12].

**Figure 3:**
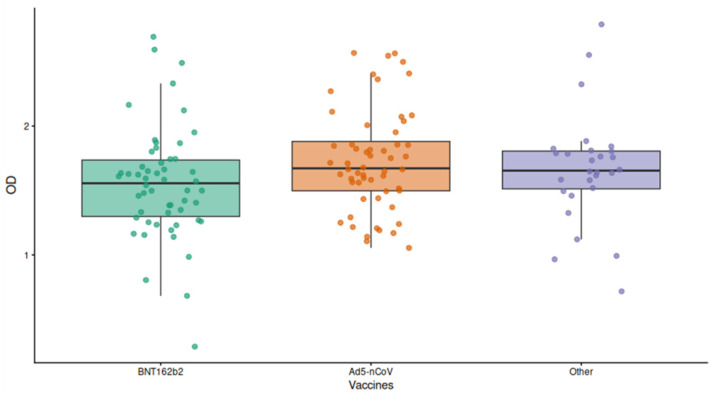
Optical density according to vaccine category. Box-and-dot plot comparing OD values among subjects vaccinated with BNT162b2, Ad5-nCoV, or other vaccines. The box indicates the interquartile range and median, while individual dots represent single observations.

The analyzed sample compared the detectable IgG response among recipients of several vaccines, including BNT162b2 and Ad5-nCoV (Figure 3), stratified according to body mass index (BMI) into three groups: healthy weight, overweight, and obesity. Detectable IgG response was assessed by calculating the percentage of positive samples within each BMI category (Table 2).

Among people maintaining a healthy weight, the Ad5-nCoV vaccine accounts for all positive samples, while the BNT162b2 vaccine accounts for 98% and other vaccines for the remaining 100%. In the overweight group, the BNT162b2 vaccine is responsible for 96% of the positive samples, followed by the Ad5-nCoVand other vaccines with 100%. Finally, individuals with obesity have 100% of positive samples attributed to the three group of vaccines (BNT162b2, Ad5-nCoVand, other vaccines).

Overall, both the Ad5-nCoV vaccine and other SARS-CoV-2 vaccines demonstrate consistently high seropositivity across all BMI categories, with nearly 100% of individuals testing positive for antibodies in most groups. Interestingly, a slight difference was observed within the Ad5-nCoV cohort, where the detectable IgG response appeared somewhat reduced among individuals with a healthy weight (91%), while those classified as overweight or obese showed a stronger response, with 98% positivity. These findings suggest that, although the general detectable antibody response of all vaccines remains high, subtle variations in their uniformity across BMI groups may exist. Such differences could reflect distinct immunological mechanisms or interactions between the type of vaccine and host metabolic factors. While these data indicate robust binding antibody production, seropositivity does not directly equate to neutralizing activity, and clinical protection cannot be inferred solely from these binding assays. This underscores the need for further functional studies to clarify these associations and fully understand vaccine-induced immunity in diverse populations.

## 3. Discussion

Our serological results demonstrated that the generation of detectable anti-SARS-CoV-2 IgG was not significantly impaired by obesity in this Mexican cohort, highlighting the broad seropositivity achieved during mass vaccination campaigns. To understand the molecular mechanisms underlying this sustained host immune response, it is crucial to analyze the structural biology of the vaccine antigens and how their rational design helps overcome the immunometabolic barriers typical of obesity. Chronic inflammation can affect the affinity of the antibodies produced, decreasing their ability to effectively neutralize the virus [13]. It has been observed that metabolic changes and chronic inflammation affect somatic hypermutation and clonal selection, processes crucial to producing high-affinity antibodies [14]. These structural and functional changes reduce antibodies’ ability to neutralize the virus, which explains why obese people may have a less effective immune response against SARS-CoV-2 [15].

The results presented here show that antibody generation was not significantly lower in the obese and overweight group compared to the healthy weight group. However, across the three BMI groups, our data support a consistently high detectable IgG response after vaccination (Figure 1). These findings are consistent with recent longitudinal evidence in vaccinated cohorts, where obesity did not consistently predict the magnitude or durability of antibody responses when controlling for confounders such as sex, prior infection, number of doses of vaccine type [16]. In that study, although some early differences in slopes of antibody production were observed depending on sex and infection history, these effects did not persist after subsequent doses, and obesity itself was not identified as a robust predictor of reduced humoral immunity. In this case, the number of doses was not considered because the present analysis was cross-sectional rather than longitudinal.

Although our cross-sectional design did not allow us to evaluate responses after completion of full vaccination schedules, evidence from other populations suggests that booster doses generally increase antibody titers in two-dose regimens such as BNT162b2 or ChAdOx1-S [17]. In individuals with obesity, some reports indicate that the subsequent decline in humoral responses may occur more rapidly. Therefore, our findings should be interpreted within the context of early and heterogeneous vaccine deployment in northern Mexico, and follow-up studies will be necessary to determine whether BMI influences the durability and functional quality of vaccine-induced immunity after full or extended regimens [17,18,19]. This makes it particularly important to assess the effectiveness of such schemes in individuals with obesity, since their altered immune profile may impact the durability of protection. This distinction underscores the need to interpret our findings within the context of heterogeneous vaccination schedules and highlights the importance of follow-up studies in obese cohorts after completion of full or extended vaccination regimens [1].

In contrast, investigations centered on natural SARS-CoV-2 infection have shown that individuals with obesity tend to produce lower levels of neutralizing antibodies and display a higher prevalence of autoantibodies when compared to lean counterparts, with these immune alterations strongly associated with systemic inflammation [20], as reflected by elevated biomarkers such as C-reactive protein. This discrepancy suggests that the immune dysregulation linked to obesity may be more evident during acute infection, when inflammatory mediators are markedly increased, than in the context of controlled antigen exposure through vaccination. Such findings underscore the importance of considering obesity not only as a metabolic condition but also as a factor that significantly shapes the quality and durability of immune responses against viral infections.

The production of antibodies is a process that can be affected by many factors like gender, age, and comorbidities [21]. Studies have reported that the generation of antibodies against SARS-CoV-2 is affected by BMI [6,20]. Differences in antibody production have been observed in populations from Australia [22], and the USA [23]. However, in the case of Mexico, the same phenomenon has not been observed (Figure 3), and no significant differences have been found as reported by the working group [11] and by Nuñez and collaborators [12].

Due to obesity, alterations in lymphoid tissue occur consequently, leading to an alteration in the coordination between innate and adaptive immunity, decreased activity of antigen-presenting cells, and lack of maintenance of specific memory B cells [24]. In a longitudinal study conducted in vaccinated healthy Japanese adults, it was revealed that the neutralizing potency and number of RBD-specific memory B cells remained stable for up to 1 year [25]. While our cross-sectional data do not allow us to evaluate the durability of the antibody response or the maintenance of memory B cells in our specific cohort, these observations from other populations suggest that obesity can negatively impact the formation and maintenance of memory B cells, which may result in less effective immune responses to recurrent infections or vaccinations and fewer antibodies produced due to a lower proliferative capacity of the cells [26].

The neutralizing capacity of antibodies relies on the structural interaction established between the antigen and the antibody, which in turn depends on prior antigenic exposure through vaccination or infection. In the case of vaccination, this interaction reflects the immune memory generated after administration. Among a cohort of 241 vaccinated individuals, 81 received the BNT162b2 vaccine and 103 received the Ad5-nCoV vaccine (Table 2), both of which employ strategies designed to induce the production of the full SARS-CoV-2 spike protein as the primary immunogen. Specifically, the BNT162b2 mRNA vaccine delivers genetic instructions that enable host cells to transiently express the complete spike protein, thereby eliciting an adaptive immune response and promoting the development of neutralizing antibodies [27]. Based on a viral vector, Ad5-nCoV vaccine uses a modified adenovirus vector, which encodes the full Spike protein of SARS-CoV-2 [28]. These two technologies are based on generating the immune response against the complete S protein (Figure 3).

For overweight individuals, whose immune responses may be partially compromised, it becomes essential to design vaccines capable of eliciting a coordinated activation of both humoral and cellular arms of the immune system. Detailed structural insights into the SARS-CoV-2 spike protein provide valuable guidance for this purpose, as they enable the rational design of vaccine candidates that optimize epitope presentation. By ensuring that the most critical antigenic sites are exposed in their most immunogenic conformations, such approaches may enhance overall vaccine efficacy and durability, even in populations with altered immune function. The robust immunogenicity observed in our cohort—despite the chronic inflammatory state associated with obesity—can be explained through this structural biology framework. Because the rational design of these vaccines ensures the optimal conformational presentation of the Spike SARS-CoV-2 protein, which forms a trimer with each monomer divided into two functional subunits (S1 and S2), they successfully trigger B-cell responses even in the altered immunometabolic environment of obese patients.

Focusing on the critical epitope that drives the induction of neutralizing antibodies has the potential to significantly strengthen the immune response. Within the SARS-CoV-2 spike protein, the RBD region stands out as the most immunodominant region, responsible for eliciting the largest proportion of antibodies. Importantly, it is also recognized as the primary target of nearly 90% of the neutralizing activity observed in sera or plasma from most individuals analyzed, underscoring its central role in protective immunity [29]. This knowledge increases the probability of generating an effective response based on the rational design of the molecule through a structural biology approach.

The RBD represents the dominant target for antibody recognition within the SARS-CoV-2 spike protein, playing a central role in shaping the adaptive immune response. Current reports indicate that more than 12,900 structural models of antibodies directed against SARS-CoV-2 have been characterized, providing valuable insights into the molecular basis of immune protection. Remarkably, over 8500 of these antibodies are specifically directed against the RBD, underscoring its immunodominance and highlighting its importance as a critical antigenic site for neutralization [30]. Spike is a highly glycosylated protein, although the glycosylation sites prevent the recognition of neutralizing antibodies [31].

The immunodominance of the SARS-CoV-2 RBD may be attributed to its relatively low degree of glycosylation compared with the rest of the spike protein, as well as to its greater accessibility on the surface of virions and infected cells. This accessibility is further increased by the opening of the RBD, which exposes otherwise cryptic epitopes [32].

The principal epitopes recognized by antibodies against the spike (S) protein, these epitopes constitute the main antigenic sites that drive the neutralizing antibody response. However, in individuals with obesity, variations may occur not only in the magnitude of antibody production but also in the overall effectiveness of the neutralizing response. Such differences could reflect alterations in immune regulation associated with metabolic status, potentially leading to reduced neutralization capacity or changes in epitope recognition patterns when compared with individuals of normal weigh [33]. Identifying and characterizing the key epitopes of the SARS-CoV-2 spike protein provides an essential framework to explore how obesity may influence the capacity of antibodies to neutralize the virus, thereby informing the development of more precise treatment and prevention strategies for this population. Importantly, emerging viral variants often present mutations that modify epitope composition and distribution on the S protein which can alter immune recognition [34].

These changes may not impact all individuals equally, as people with obesity could experience distinct alterations in neutralization efficiency due to their altered immunological profile. A deeper understanding of how such variants differentially affect neutralizing responses in individuals with obesity will be critical for tailoring vaccine formulations and optimizing protective strategies in diverse populations. While the frequency of infections was not included in our analysis, the structural dynamics of the spike epitopes presented here provide a basis to hypothesize how antibody responses might influence reinfection or breakthrough cases. Mapping these structural changes is essential before integrating epidemiological data in follow-up studies [35].

With the emergence of new variants, the RBD has become a critical region to study, as most mutations occurring within it modify either charge or hydrophobicity. These alterations substantially increase the likelihood of antibody escape, either by changing epitope affinity or by inducing local conformational shifts that reduce epitope accessibility [36].

The use of rational design for therapy design based on structural biology approaches has greater advantages compared to genetic engineering, especially in people with obesity. This approach uses detailed information about the structure of viral proteins, which can lead to more specific and effective therapies, for those who may have compromised or different immune responses than people of normal weight.

In this study, we examined the immune response in overweight populations, including the complete structure of the protein, the most immunodominant regions, and the epitopes that interact most with neutralizing antibodies. Nevertheless, it is important to clarify that the ELISA used in this study was a semiquantitative assay; therefore, it was not possible to measure the precise antibody titers induced by vaccination, but only to assess the response in a qualitative or semiquantitative manner. Importantly, previous studies have demonstrated that semiquantitative ELISA assays show a strong correlation with neutralizing antibody titers, supporting their use as reliable proxies of protective immunity [37]. However, interpreting these binding assays requires caution, as our cross-sectional design cannot confirm long-term protection, and without functional assays, the structural biology framework discussed remains a hypothesis-generating perspective. Furthermore, our analysis did not control for prior SARS-CoV-2 infection; this represents a major confounder, as hybrid immunity (infection and vaccination) can substantially increase antibody levels, seropositivity rates, and neutralization capacity. This limitation acquires greater relevance when considering the lack of post-vaccination immunological data reported from Latin American countries. While numerous studies have evaluated antibody responses in high- and middle-income countries, evidence from Latin America remains scarce, particularly when generated using methodologies adapted to local resource constraints.

Under these conditions, semiquantitative analyses such as ours provide essential information that helps delineate immune responses in Mexican cohorts, filling a critical regional gap and contributing to a broader understanding of vaccine effectiveness [38].

## 4. Materials and Methods

### 4.1. Study Participants

This study recruited 138 individuals aged 18 years and older from the cities of Reynosa and Matamoros, Tamaulipas, Mexico. The cohort had a median age of 42.0 years (interquartile range [IQR]: 27.5–51.0), and the sex distribution was 58.7% female (*n* = 81) and 41.3% male (*n* = 57). At the time of sampling (June 2021–June 2022), due to national public health strategies prioritizing initial coverage amidst vaccine scarcity, all participants in our cohort had received only a single primary dose of a SARS-CoV-2 vaccine. The complete vaccination schedule was not yet available during that period. Consequently, the exact time elapsed from vaccination to blood sampling varied widely among participants and could not be standardized, reflecting a real-world, cross-sectional scenario during the early deployment of vaccines in northern Mexico.

### 4.2. Ethics Approval and Informed Consent

Participants completed a questionnaire approved by the Research Ethics Committee of the School of Medicine of the Universidad de Monterrey-Nuevo Leon (protocol code 05/2021; date of approval 2 May 2021); containing clinical and demographic information, including history of SARS-CoV-2 infection, documented evidence of the type of vaccine administered, vaccination schedule and dosage, vaccine-related adverse events, sex, age, weight, height, and the presence or absence of comorbidities.

### 4.3. The Enzyme-Linked Immunoassay (ELISA)

IgG antibodies against SARS-CoV-2 were detected using the V2G^®^ kit (UDIBI, Ciudad de México, Mexico), provided by the Unidad de Desarrollo e Investigación en Bioterapeuticos (UDIBI) at the Instituto Politécnico Nacional, Mexico City. This ELISA-based method identifies human serum IgG antibodies against SARS-CoV-2 with a sensitivity of 99.33% and a specificity of 97.82%. Optical density (OD) was measured at 450 nm using a Varioskan LUX (Agilent Technologies, Santa Clara, CA, USA) microplate reader with SkanIt Software 6.0. 6.0.0.44 (Thermo Fisher Scientific, Waltham, MA, USA). Interpretation of results followed UDIBI’s technical specifications: an OD ≤ 0.5 indicated a negative result (no detectable IgG antibodies against SARS-CoV-2), whereas an OD ≥ 0.6 indicated a positive result (presence of IgG antibodies against SARS-CoV-2). The experimental methodology was developed as reported briefly by Rodríguez-Martínez et al. [11].

### 4.4. Statistical Analysis

Second BMI were calculated, and participants were divided into three groups according to OMS criteria, the first group (18.5–24.9 kg/m^2^) was defined as healthy weight, the second group (25–29.9 kg/m^2^) was defined as overweight, and the last group (BMI ≥ 30 kg/m^2^) was defined as having obesity. Antibody levels were defined as the optical density values obtained from the post-immunoassay reading, both below and above the established cut-off threshold. To evaluate the effect of body mass index (BMI) on the generation of antibodies, Kruskal–Wallis’s test was performed. The Wald method was used to estimate the SARS-CoV-2 IgG antibody generation rate in the sample population and the adjusted confidence intervals (CIs) surrounding the point estimates. Statistical analyses were performed with R studio (version 4.2.2).

## 5. Limitations

An important limitation of this study is that it includes participants who received only a single dose of the COVID-19 vaccine. Due to vaccine scarcity and prioritization strategies in Mexico during the sampling period, the complete vaccination schedule was not widely available. This single-dose regimen may impact the generalizability of our findings to fully vaccinated or boosted populations and should be carefully considered when interpreting the magnitude and durability of the humoral immune response. Additionally, as previously mentioned, our cross-sectional design cannot confirm long-term protection, and our analysis did not control for prior SARS-CoV-2 infection, which is a major confounder.

## 6. Conclusions

In this study, we evaluated the impact of obesity on the generation of anti-SARS-CoV-2 IgG antibodies in a Mexican cohort and found no significant quantitative differences across BMI groups. These results demonstrate that mass vaccination campaigns can successfully elicit robust host immune responses even in populations with a high prevalence of metabolic dysfunction. Furthermore, our findings contrast with previous reports from other regions and provide a critical rationale for rethinking how we interpret vaccine efficacy in Latin America. While obesity did not impair the overall quantity of antibodies in our cohort, the molecular mechanisms underlying virus neutralization—such as epitope accessibility, antibody affinity, and dynamic interactions with the Spike protein—may still be influenced by the host’s immunometabolic state. Therefore, approaching future epidemiological and immunological studies from a structural biology perspective will be essential to fully unravel the molecular basis of immune protection and to design tailored therapeutic interventions for populations with distinct genetic and metabolic backgrounds.

## Figures and Tables

**Table 1:** Distribution of total samples (*n* = 138) and positive cases (*n* = 137) according to body mass index (BMI). Calculations of the antibody rate by BMI were based exclusively on positive samples, and the corresponding adjusted Wald 95% confidence intervals (CIs) are reported for each point estimate.

Variable	Total Samples		Positive Samples		Antibody Rate	ICs	Kruskal–Wallis
	*n* = 138	%	*n* = 137	%	%	95%	*p*-Value
BMI							
Healthy weight	32	23.2	32	100	24	16–30	
Overweight	60	43.5	59	98.3	43	34–51	0.20
Obesity	46	33.3	46	100	33	26–41	

**Table 2:** The number of samples of each vaccine within BMI variable. Among others are ChAdOx1-S, CoronaVac, mRNA-1273 and Ad26.COV2.S.

			Vaccines			
Variable	BNT162b2	Positive Samples	Ad5-nCoV	Positive Samples	Others	Positive Samples
	*n* = 54	%	*n* = 56	%	*n* = 28	%
BMI						
Healthy weight	15	100	8	100	9	100
Overweight	24	96	26	100	10	100
Obesity	15	100	22	100	9	100

## Data Availability

Dataset available on request from the authors.
